# Passage-Based Bibliographic Coupling: An Inter-Article Similarity Measure for Biomedical Articles

**DOI:** 10.1371/journal.pone.0139245

**Published:** 2015-10-06

**Authors:** Rey-Long Liu

**Affiliations:** Department of Medical Informatics, Tzu Chi University, Hualien, Taiwan, R. O. C; Katholieke Universiteit Leuven, BELGIUM

## Abstract

Biomedical literature is an essential source of biomedical evidence. To translate the evidence for biomedicine study, researchers often need to carefully read multiple articles about specific biomedical issues. These articles thus need to be highly related to each other. They should share similar core contents, including research goals, methods, and findings. However, given an article *r*, it is challenging for search engines to retrieve highly related articles for *r*. In this paper, we present a technique PBC (Passage-based Bibliographic Coupling) that estimates inter-article similarity by seamlessly integrating bibliographic coupling with the information collected from *context passages* around *important out-link* citations (references) in each article. Empirical evaluation shows that PBC can significantly improve the retrieval of those articles that biomedical experts believe to be highly related to specific articles about gene-disease associations. PBC can thus be used to improve search engines in retrieving the highly related articles for any given article *r*, even when *r* is cited by very few (or even no) articles. The contribution is essential for those researchers and text mining systems that aim at cross-validating the evidence about specific gene-disease associations.

## Introduction

Biomedical literature has accumulated a huge and ever-increasing amount of biomedical evidence. To translate the evidence for biomedicine study, researchers routinely spend much effort retrieving the evidence from literature. Given a specific issue (e.g. association among specific genes, diseases, chemicals, and proteins), the researchers need to carefully read multiple articles to exclude controversial evidence about specific issues. For example, to maintain a database of gene-disease associations, Genetic Home Reference (GHR) recruits hundreds of curators that carefully check multiple articles [1] and routinely update the database in each week [2]. Curators of Online Mendelian Inheritance in Human (OMIM) even update their database on a daily basis [3].

Therefore, it is essential to retrieve related articles for the biomedical researchers [4]. One way to retrieve the articles is to set a query about specific biomedical entities (e.g., genes and diseases) and then search for those articles that are related to the query [5]. Another way is to designate an article *r*, and then retrieve those articles that are related to *r*. Therefore, several search engines (e.g., PubMed) have provided the service of recommending related articles for a given article (the way of computing the similarity score by PubMed can be found in [6, 7]). However, they do not aim at recommending *highly related* articles. Highly related articles should share similar *core contents*, including research goals, methods, and findings. By reading the highly related articles, the researchers can focus their limited effort on checking specific issues. It is challenging to retrieve highly related articles, as it is quite difficult to recognize the core contents of biomedical articles.

**Fig 1:**
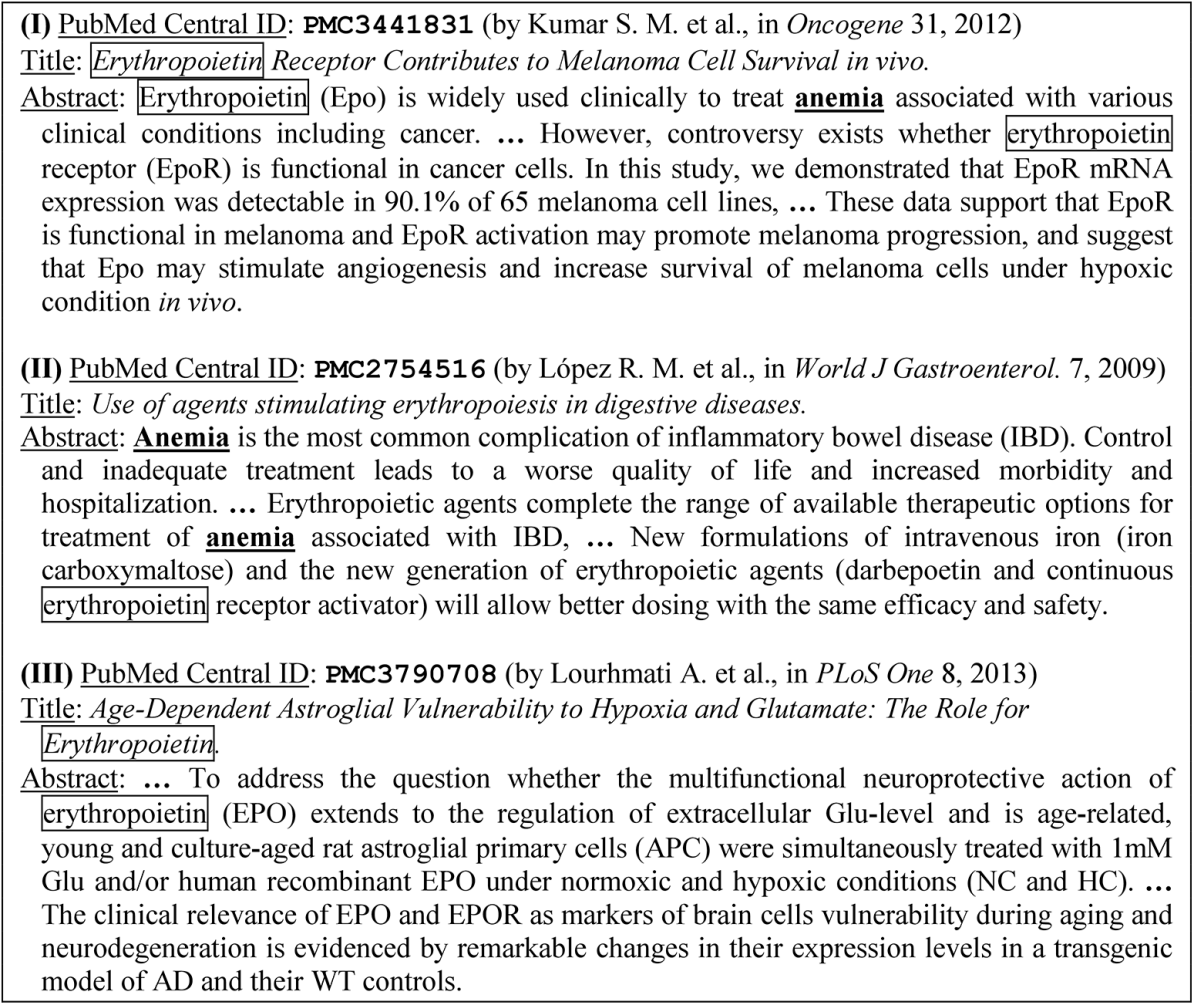
Three articles (excerpted) to illustrate the challenge of retrieving highly related biomedical articles. The first two articles were selected by biomedical experts to curate the association between erythropoietin (marked in boxes) and anemia (boldfaced and underlined), but the third article does *not* focus on the association. The first two articles are thus highly related to each other, although they do not share any out-link citations and PubMed does not recommend them as related articles.

In this paper, we present a technique that, given a biomedical article *r* as the target, retrieves highly related articles for *r*. The technique is based on *bibliographic coupling* [8], which expects that two articles *d*1 and *d*2 may be related to each other if they cite a similar set of articles (i.e., *d*1 and *d*2 share similar *out-link* citations or references). However, bibliographic coupling has an essential weakness in identifying highly related articles, as two highly related articles about a research issue *x* may cite *different* articles whose core contents are all about *x*. For example, consider three example articles shown in Fig 1. The first two articles (PMC3441831 and PMC2754516) are obtained from DisGeNET (available at www.disgenet.org/web/DisGeNET/v2.1/home), which maintains a database of articles that biomedical experts selected to annotate individual gene-disease associations. The first two articles were selected by the biomedical experts to curate the association between erythropoietin and anemia, and hence they are highly related to each other. However they do *not* share any out-link reference, and given the first article (PMC3441831), PubMed did *not* recommend the second article (PMC2754516) as a related article (we used the “Related citations in PubMed” service to check the related articles recommended by PubMed in January 2015). On the other hand, the 3^rd^ article (PMC3790708) does *not* focus on the association between erythropoietin and anemia, and hence it was *not* selected by the biomedical experts for the association. However given the first article (PMC3441831), PubMed recommended the 3^rd^ article as a related article. Therefore, both bibliographic coupling and PubMed have weaknesses in retrieving highly related articles for a given article. We aim at tacking the weaknesses.

We thus propose a novel technique PBC (Passage-based Bibliographic Coupling), which seamlessly integrates bibliographic coupling with the information collected from *context passages* of *important out-link* citations (references) in each article. The context passage of a reference *c* in an article *d* is the text (in *d*) that the authors of *d* employ to explain why *c* is related to *d*. Therefore, two articles *d*1 and *d*2 that cite two different references *c1* and *c2* respectively may be related to each other if *c1* and *c2* have similar context passages in *d*1 and *d*2. The context passages can thus be employed to tackle the weakness of bibliographic coupling. We will show that PBC performs significantly better than several state-of-the-art techniques that the ones that individually consider link-based or text-based information, as well as a hybrid technique that considers both link-based and text-based information.

## Related Work

Previous techniques for inter-article similarity estimation fall into three types: (1) *text-based* techniques, (2) *link-based* techniques, and (3) *hybrid* techniques. Text-based techniques estimated the similarity between two articles based on how the two articles share certain textual contents, while link-based techniques estimated the similarity based on how the two articles share certain out-links (i.e., they cite the same articles) and/or in-links (i.e., they are cited by the same articles). Hybrid techniques employed both text-based and link-based information to estimate inter-article similarity.

### Text-based techniques

Text-based techniques often considered the *weights* of the terms shared by two articles to estimate the similarity between the two articles. The vector space model (VSM) is a typical text-based technique. It represents an article as a vector of term weights and employs the cosine similarity to estimate inter-article similarity. VSM was employed by many retrieval systems such as Lucene (available at http://lucene.apache.org), which was employed in many biomedical text retrieval and mining studies (e.g., [9]).

As the cosine similarity did not perform quite well in many cases [10], other similarity estimation techniques were developed and/or tested [10–11], and their main ideas were employed by PubMed, which was found to be one of the best systems in finding related biomedical articles [11]. PubMed assigns higher weights to those terms that appear at certain positions in the articles (e.g., titles, abstracts, and biomedical keywords of the articles), as well as those terms that appear infrequently in biomedical literature (i.e., rare terms). The similarity score is higher if the two articles share many terms with higher weights [6, 7]. Similar ideas were routinely employed by previous studies (e.g., [12]) and used to develop better techniques (e.g., [10]).

However, the text-based techniques can only measure how terms co-occur in both articles, and hence cannot properly measure the similarity between the core contents of the two articles. This is also a reason why text-based techniques often considered certain key parts of the articles only (e.g., titles and abstracts of the articles, as done by PubMed). Although the key parts of an article *r* tend to present the main idea of *r*, they are often too short to contain all the core content of *r*, which should be a *complete* description of the research goals, research methods, and main findings of *r*. Therefore, our proposed technique PBC considers citation links to estimate inter-article similarity. It is based on bibliographic coupling. We will show that PBC can perform significantly better than those text-based techniques that were found to be the best ones by [10–11].

### Link-based techniques

Link-based techniques considered citation links among articles. We are concerned with those link-based techniques that aimed at estimating *inter-article similarity* (some previous techniques aimed at different purposes such as identifying important biomedical articles [13] and expanding the query to retrieve biomedical articles [14]). To estimate inter-article similarity, previous linked-based techniques employed two kinds of citation links: *in-links* and *out-links*. *Co-citation* [15] is a representative technique that considers in-links. It is based on the expectation that two articles *d*1 and *d*2 may be related to each other if they are co-cited by other articles (i.e., *d*1 and *d*2 share similar in-link citations). Co-citation was found to be useful in classifying webpages [16–17] and measuring the professional similarity between authors [18]. However, it is quite difficult to collect in-link citations, due to two reasons: (1) many research articles have very few (or even no) in-link citations (especially those articles that were recently published), and (2) it is difficult to collect a complete set of in-link citations for a given article. *Out-link* citations were thus shown to be more helpful than in-link citations in the classification of research articles [16].

Therefore, many previous studies employed out-link citations to estimate inter-article similarity, and PBC employs out-links as well. Bibliographic coupling is a representative technique that considers out-links. Eq 1 is a typical way to estimate bibliographic coupling similarity (BC) between two articles *d*1 and *d*2 [16–17], where *O*_d1_ and *O*_d2_ are the sets of articles that *d*1 and *d*2 cite respectively (i.e., out-link references in *d*1 and *d*2 respectively). When both *O*_d1_ and *O*_d2_ are empty, BC is set to 0.

$$BC(d1,d2)=\frac{\left|O_{d1}\cap O_{d2}\right|}{\left|O_{d1}\cup O_{d2}\right|}$$ (1)

Bibliographic coupling was shown to be useful in several applications, including the classification of scientific papers [16], retrieval of similar legal judgments [19], detection of plagiarism [20], and measurement of the professional similarity between authors [21]. It was extended by considering the common articles (entities) that were cited (linked) by *d*1 and *d*2 *indirectly* [22]. However, as noted above, bibliographic coupling has an essential weakness, as two highly related articles about a research issue *x* may cite different articles whose core contents are about *x*. Our proposed technique PBC tackles the weakness by employing the *context passages* around *important* out-link references. PBC is thus a hybrid technique that considers both link-based and text-based information. We will show that PBC perform significantly better than bibliographic coupling, which had competitive performance when compared with several hybrid techniques that considered both text-based and link-based information [16, 23].

### Hybrid techniques

Hybrid techniques consider both text-based and link-based information. The link-based information can be the mean age of out-link references and the percentage of the references to serials [24], rather than the citation relationships between articles. The goal of the hybrid techniques can be to aggregate cross citations to classify or cluster journals [25–26], rather than specific articles. These hybrid techniques thus have goals different from the goal of PBC: exploring the potential contribution of integrating text-based information and out-link citations to retrieve highly related articles. We thus focus more on comparing PBC with those hybrid techniques that estimated inter-article similarity by text-based information and citation relationships.

Many previous hybrid techniques employed *in-link* citations (rather than out-link citations). For example, proximity of two citations in the full texts of the articles was employed to estimate inter-article similarity (i.e., two articles *d*1 and *d*2 may be more related to each other if they are cited in a nearby area in a citing article *d* [27, 28]). Context passages around citations was employed to enhance the representation of articles, with the goal to improve topic-based article retrieval [29, 30] or inter-article similarity estimation [31]. These hybrid techniques were based on the expectation that, for a given article *x*, the context passages in the articles that cite *x* may indicate the main contents of *x* [32], although different citing articles may focus on different aspects of *x* [19, 33] with different sentiments [34]. In the bioscience domain, the in-link context passages were believed to be helpful for article summarization and retrieval, as well as the recognition and disambiguation of biomedical named entities [35]. However, as noted above, applicability of these in-link-based techniques is limited, because many articles have very few (or even no) in-link citations and it is difficult to collect a complete set of in-link citations for a given article. PBC is a hybrid technique that relies on out-links citations to estimate inter-article similarity.

Several previous hybrid techniques employed out-link citations as well. Some studies reported that the hybrid techniques did not necessarily perform significantly better than the techniques based on bibliographic coupling, even when full-text articles and text classifiers were employed [16, 23]. Some studies reported that the hybrid techniques could perform better. Bibliographic coupling similarity and full-text similarity were integrated to cluster articles [36]. However, the performance heavily depended on the evaluation criteria: link-based (hybrid) evaluation criteria tended to favor link-based techniques (hybrid techniques). Therefore, to measure the contributions of a hybrid technique more properly, the evaluation criterion should be independent of texts and citations [37]. Our evaluation criterion is based on semantic relatedness: it checks whether the systems can retrieve those highly related articles that are judged by biomedical experts. A more recent work employed the relatedness between biomedical articles as an evaluation criterion as well [37], without relying on co-words and co-citations to design the criterion. It integrated bibliographic coupling similarity with text-based similarity by treating a co-word in two articles as a citation co-cited by the two articles. This integrated similarity measure (named *HybridK50*) was tested in the biomedical domain as well, and performed better than bibliographic coupling [37]. We thus implement *HybridK50* as a baseline, and will show that PBC performed significantly better than *HybridK50*.

Therefore, when compared with the previous studies, technical contributions of PBC are to seamlessly integrate bibliographic coupling with the information collected from the *context passages* around *important out-link* citations, which are available in articles. We will show that PBC performs better than several state-of-the-art techniques, including bibliographic coupling, two text-based techniques, and a hybrid technique. In practice, the highly related articles recommended by PBC are helpful in several cases: (1) for *readers* and *curators* of an article *r*, the highly related articles can support more timely and complete analysis of related evidence, even though the evidence was published recently; (2) for *authors* of an article *r*, the articles that are highly related to *r* can facilitate more complete literature survey; (3) for *reviewers* of an article *r*, the highly related articles are helpful for the reviewers to verify the contribution of *r* and check the possible problem of plagiarism; and (4) for *text mining systems* that work on an article *r*, the highly related articles facilitate more focused mining of related evidence.

## Passage-Based Bibliographic Coupling

For an article *r*, the input to PBC includes the title of *r*, the out-link citations (references) of *r*, and the passages around the places where the references are discussed in *r*. PBC has three challenges: (1) extracting the *context passage* of each reference in *r*, (2) estimating the *degree of importance* for each reference (in *r*) based on likelihood of the reference being related to the core contents of *r*, and (3) estimating the *similarity* between two articles based on the context passages and the degrees of importance.

The context passage of a reference *c* in an article *d* consists of two parts: (1) the title of *d* and (2) the text immediately before each place *p* where *c* is mentioned in the main body of *d*. Both parts can provide strong information about how the author(s) of *d* discussed *c* from the perspective of the core contents of *d*. The former can indicate the general context of the discussion, as the title indicates the goal of *d*. The latter can indicate the specific issue of the discussion, as the text immediately before *p* should provide specific comments on *c*. More specifically, after removing stopwords from *d*, the context passage (CP) of *c* in *d* is extratced by Eq 2.

$$CP(c,d)=\cup \left\{words\;in\;title\;of\;d\right\}\;\cup \underset{\begin{array}{l} p\in \{places\;where\;\\ c\;appears\;in\;d\} \end{array}}{\cup }\left\{\alpha \;words\;before\;p\right\}$$ (2)

Eq 2 has a parameter α (alpha) that governs the number of words preceeding the place where *c* is mentioned in *d*. We hypothesize that the parameter should be around 10, as authors of research articles tend to comment a citation with a short passage. In the experiment, we will investigate the hypothesis, and show that properly setting the parameter is not a difficult task. Also note that, instead of employing complicated techniques to recognize the context passages (e.g., considering certain linguistic patterns around citations [38]), Eq 2 considers the words *before c* is mentioned in *d*, because this method is efficient, and the text used to comment a citation tends to appear before the citation. In the experiment, we will show that this method is helpful in improving the retrieval of related articles.

Given the context passage of each reference, PBC employs Eq 3 to estimate the similarity betwen two references *c*1 and *c*2 in articles *d*1 and *d*2 respectively. The context passgaes of *c*1 and *c*2 are helpful when *c*1 and *c*2 are not identical but they are respectively commented by the authors of *d*1 and *d*2 with similar words. The similarity falls in the range of [0, 1].

$$LinkSim(c1,d1,c2,d2)=\{\begin{array}{l} \begin{array}{l} \;1,\;if\;c1=c2; \end{array}\\ \;\frac{\left|CP(c1,d1)\cap CP(c2,d2)\right|}{\left|CP(c1,d1)\cup CP(c2,d2)\right|},\;otherwise \end{array}$$ (3)

We are also concerned with the *importance* of each reference. A reference *c* in *d* is more important in *d* if it is more related to the core content of *d*. Generally, a reference may be discussed for different purposes, such as (1) describing the research background and tools, (2) discussing related but different studies, and (3) comparing baseline approaches or ideas. Those references that are discussed for the latter two purposes should be more important, and they tend to be discussed in an article more than one time. Therefore, the degree of importance (*IMP*) of a reference *c* in an article *d* is defined in Eq 4.

$$IMP(c,d)=\{\begin{array}{l} \begin{array}{l} \;2,\;if\;c\;appears\;at\;least\;two\;times\;in\;d; \end{array}\\ \;1,\;otherwise \end{array}$$ (4)

By integrating the link similarity (Eq 3) and the link importance (Eq 4), we have Eq 5, which is the weighted similarity between two references. The similarity falls in the range of [0, 2], as *LinkSim* and *IMP* fall in the range of [0, 1] and [1, 2], respectively.

$$LinkSimIMP(c1,d1,c2,d2)=LinkSim(c1,d1,c2,d2)\times \frac{IMP(c1,d1)+IMP(c2,d2)}{2}$$ (5)

With the way to estimate the weighted similarity between two references, PBC estimates the similarity between two articles *d1* and *d2* by Eq 6, where *O*_*d1*_ and *O*_*d2*_ are the sets of references in *d1* and *d2* respectively. The similarity falls in the range of [0, 2], as *LinkSimIMP* falls in the range of [0, 2]. PBC employs the similarity values to rank articles.

$$PBC(d1,d2)=\frac{\sum_{c1\in O_{d1}}Max_{c2\in O_{d2}}LinkSimIMP(c1,d1,c2,d2))+\sum_{c2\in O_{d2}}Max_{c1\in O_{d1}}LinkSimIMP(c1,d1,c2,d2))}{\left|O_{d1}|+|O_{d2}\right|}$$ (6)

Therefore, given two articles *d1* and *d2*, PBC produces a large similarity value for them if important references in *d1* and *d*2 share similar context passages. In such case, *d1* is said to be more similar to *d2* even if they have no out-link citations in common. Moreover, PBC does not rely on any in-link citations of the articles, and hence is suitable for the common case where articles have very few (or even no) in-link citations.

We are concerned with the time required to compute the inter-article similarity. To estimate the similarity between the two articles *d*1 and *d*2, PBC estimates the similarity between each possible *c*1 and *c*2, where *c*1⊰*O*_d1_ and *c*2⊰*O*_d2_. PBC thus requires |*O*_d1_|×|*O*_d2_| computations, which should be acceptable in practice, because (1) the number of out-links in an article (i.e., |*O*_d1_| and |*O*_d2_|) is often not quite large, and (2) the computations are done only once, with the results cached for use online.

We are also concerned with different strategies to estimate the importance of each reference in an article. In addition to the frequency-based approach to estimating the importance (i.e., IMP in Eq 4), we also implement two strategies as the baselines for performance comparison with PBC: the position-based strategy and the section-based strategy. In the position-based strategy, a reference c is more important in an article d if it is cited at the place near to the end of d. This strategy is based on the expectation that important references tend to appear in the main results and discussions (in d), which tend to appear at the places near to the end of d. More specifically, the position-based importance is defined in Eq 7, and PBC with the position-based strategy is named PBC-Pos.

$$IMP_{Pos}(c,d)=1+Max_{p\in \{Positions\;of\;c\;in\;d\}}\frac{p}{Length(d)}$$ (7)

On the other hand, in the section-based strategy, a reference is more important in an article d if it is cited in or after the result section in d. This strategy is based on the expectation that important references tend to be discussed in the sections about results and discussions in d. More specifically, the section-based importance is defined in Eq 8, and PBC with the section-based strategy is named PBC-Section. A similar strategy was tested by [14] as well, although it was used for expanding the query to retrieve biomedical articles rather than estimating inter-article similarity. A section is recognized as a result section if its title has the term ‘Result’.

$$IMP_{\mathrm{Sec}tion}(c,d)=\{\begin{array}{l} \begin{array}{l} \;2,\;if\;c\;appears\;in\;or\;after\;the\;result\;\sec tion\;of\;d; \end{array}\\ \;1,\;otherwise \end{array}$$ (8)

Employing *PBC-Pos* and *PBC-Section* as the baselines is motivated by a finding of many previous studies: passages in certain places of biomedical articles tend to indicate more significant information. For example, to extract the information about the functions of genes, previous studies preferred those passages that appear at certain positions in the articles [39, 40], and to identify articles about specific entities (chemicals, genes, or diseases), previous systems preferred those abstracts in which the entities appeared at certain positions (e.g., the titles, the first sentences, and the last sentences of the abstracts, [9, 41]). Although the previous studies did not aim at retrieving highly related articles for a given article, their ideas should be tested in the experiment so that the contribution of PBC can be evaluated more completely.

## Experiments

To investigate the performance and contribution of PBC in real-world scenarios, two experiments on real-world data are conducted. Table 1 summarizes the main settings for the experiments, which are described in the next subsections.

**Table 1:** Main settings for two experiments (Experiment I and Experiment II).

Items	Experiment I	Experiment II
(1) Experimental data	Collect gene-disease pairs, and for each pair, collect two kinds of biomedical articles: (A) *Highly related biomedical articles*: For each gene-disease pair <*g*,*d*>, collect the biomedical articles that biomedical experts selected to annotate the pair; (B) *Near-miss biomedical articles* (Non-highly related articles): For each gene-disease pair <*g*,*d*>, collect articles using two queries: “*g* NOT *d*” and “*d* NOT *g*”. These articles are thus *not* highly related to <*g*,*d*> as they mention *g* or *d* but not both.	
(2) Baselines for performance comparison	(A) *Link-based* technique: *Bibliographic coupling* (BC, see Eq 1), which is a representative technique that considers *out-link* references; (B) *Text-based* technique: A similarity measure *OK* (see Eq 9), which is a state-of-the-art technique based on BM25; (C) *Hybrid* technique: A similarity measure *HybridK50*, which integrates both link-based similarity and text-based similarity	The “*Related Citations*” service provided by PubMed, which is an essential search engine in the biomedical domain: To estimate inter-article similarity, PubMed considers several typical factors, including (1) article length, (2) word recognition and stemming, (3) word positions, (4) key terms of the articles in biomedical thesauri, and (5) word weights.
(3) Evaluation criteria	Two criteria to measure how highly related articles are ranked high: (A) *Mean Average Precision* (MAP); (B) *Average precision at top-X* (Average P@X), with X = 1, 3, and 5.	

### The data

The data needs to consist of a number of articles that biomedical experts identified as *highly related* to each other. Therefore, we extract gene-disease pairs from DisGeNET (available at www.disgenet.org/web/DisGeNET/v2.1/home), which is a platform that collects and integrates information about hundreds of thousands of gene-disease associations from several data sources and the biomedical literature. For each gene-disease pair, DisGeNET lists the PubMed articles that are closely related to the pair. We select those gene-disease pairs that fulfill two requirements: (1) they have the largest number of related PubMed articles among which at least two are included PubMed Central (PMC, available at http://www.ncbi.nlm.nih.gov/pmc/), which provides the full texts of the articles; and (2) the PubMed articles are selected by GAD (Genetic Association Database, available at http://geneticassociationdb.nih.gov) or CTD (Comparative Toxicogenomics Database, available at http://ctdbase.org) for human. Both GAD and CTD have been developed to serve the scientific community for more than ten years. Biomedical experts were recruited to select articles to annotate specific gene-disease pairs [41–42]. For CTD, doctoral-level curators were trained to employ controlled vocabularies to select the articles, and the curators achieved good curation accuracy with a high degree of inter-curator agreement in an experiment [41]. The articles selected for a gene-disease pair can thus be expected to be highly related to each other. We totally obtain 53 gene-disease pairs in the experiment.

**Fig 2:**
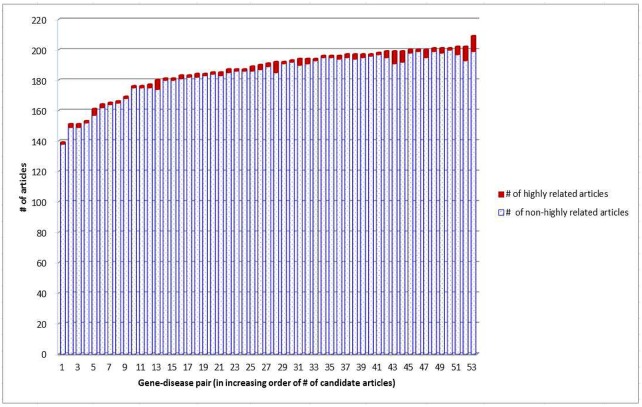
Distribution of the data. For each gene-disease pair, an article selected by biomedical experts is designated as the target article. The average percentage of articles that are highly related to the target articles is 1.34%.

For each gene-disease pair, we select the most recently published article as the *target* article, and the other articles as the *candidate* articles that are highly related to the target. Moreover, in practice a search engine often needs to rank a large number of candidate articles that are *not* highly related to the target but share some contents with the target. Therefore, for each gene-disease pair <*g*, *d*>, we send two queries to PMC: “*g* NOT *d*” and “*d* NOT *g*”, which retrieves at most 200 “near-miss” candidate articles (as they mention *g* or *d* but not both). These near-miss articles are thus *not* highly related to <*g*, *d*> although they mention *g* or *d*. A better retrieval system should rank higher (lower) those candidate articles that are highly related (near-miss) to the target article. After removing those articles that have no out-link references, we totally have 53 target articles, as well as 9,876 candidate articles among which 135 are highly related to the target articles for individual gene-disease pairs. The PMC IDs of these articles are archived in S1 File. These articles totally have 435,786 out-link references (i.e., on average an article cites 43.89 references). Fig 2 shows the distribution of the highly related articles and the near-miss articles in each gene-disease pair. The average percentage of highly related articles in the pairs is 1.34%. Identification of these highly related articles is thus challenging, especially because the near-miss articles tend to share many terms with the target articles.

A few steps are conducted to preprocess the articles, including dealing with regular gerunds (e.g., replacing 'working' with 'work') and plurals (e.g., replacing 'works' with 'work'), removing ‴s" from the end of each word, transforming characters into lowercase characters, and then removing stopwords. No multi-word terms or phrases are considered. PBC and all the baselines work on the same set of preprocessed articles.

### Baselines for performance comparison with PBC

To evaluate PBC with respect to representative inter-article similarity estimation techniques, we design two experiments (Experiment I and Experiment II) in which PBC is compared with *link-based*, *text-based*, and *hybrid* techniques. The link-based baseline is bibliographic coupling. The text-based baselines include a state-of-the-art inter-article similarity measure *OK* [10] and the “Related Citations” service provided by PubMed, which was shown to be one of the best systems in finding related biomedical articles [11]. The hybrid baseline is a technique (named *HybridK50*) that achieved better performance by integrating bibliographic coupling with text-based information [37].

In Experiment I, we aim at investigating the technical contribution of PBC, and hence we employ bibliographic coupling, *OK*, and *HybridK50* as the baselines. Bibliographic coupling (ref. Eq 1) considers out-link references to estimate inter-article similarity. It had competitive performance when compared with several hybrid techniques that considered both text-based and link-based information [16, 23]. PBC retrieves highly related articles based on *out-link* references as well, which is motivated by the fact that most research articles get very few (and even no) in-link citations and it is quite difficult to collect the in-links citations for an article. Therefore, those previous techniques that relied on in-link citations are not employed as the baselines, and with bibliographic coupling as a link-based baseline, we can evaluate how PBC employs context passages of important out-link references to improve the retrieval of related articles.

The text-based baseline *OK* was shown to be one of the best techniques to identify related articles [10]. It was based on Okapi BM25 [43], which was found to be one of the best techniques in finding related biomedical articles [11]. More specifically, *OK* estimates the similarity between two articles *d*_1_ and *d*_2_ by Eq 9.

$$OK(d_{1},d_{2})=\sum {}_{t\in d_{1}\cap d_{2}}\frac{TF(t,d_{1})(k_{1}+1)}{TF(t,d_{1})+k_{1}(1-b+b\frac{\left|d_{1}\right|}{avgdl})}\frac{TF(t,d_{2})(k_{1}+1)}{TF(t,d_{2})+k_{1}(1-b+b\frac{\left|d_{2}\right|}{avgdl})}Log_{2}\frac{N}{n}$$ (9)

In Eq 9, N is the total number of articles, *n* is the number of articles containing *t*, *k*_1_ and *b* are respectively set to 8 and 1.0 (as suggested by [10]), *|d*| is the number of words in article *d* (i.e., length of *d*), *avgdl* is the average number of words in an article, and TF(*t*,*d*) is the frequency of term *t* in article *d*. We implement two versions for *OK*: *OK-TitleAbstract* and *OK-WholeArticle*. The former works on titles and abstracts of the articles only, while the latter works on all contents of the articles. As noted above, text-based techniques often expect that certain key parts of the articles may be more informative (e.g., PubMed works on titles and abstracts of articles). Therefore, with the two versions for *OK*, we can investigate the contribution of PBC more completely.

The hybrid technique *HybridK50* is employed as a baseline because it was a hybrid technique that performed better than bibliographic coupling in identifying related biomedical articles as well [37]. *HybridK50* integrates bibliographic coupling with text-based information by treating a co-word in two articles as a citation co-cited by the two articles. *HybridK50* similarity between two articles *d*1 and *d*2 is defined based on the intersection in words and out-link citations. It is a “relative” similarity measure in the sense that it is defined by subtracting the expected degree of intersection from the actual degree of intersection. For more detailed definition of *HybridK50*, we refer the reader to [37]. We implement two versions: *HybridK50-TitleAbstract* and *HybridK50-WholeArticle*. The former works on titles and abstracts of the articles only, while the latter works on all contents of the articles.

In Experiment II, we aim at investigating the practical contribution of PBC, and hence the “Related Citations” service provided by PubMed is employed as the baseline. PubMed is an essential search engine for biomedical researchers. It employs a text-based technique to estimate inter-article similarity. Several previous studies employed the service in their experiments as well (e.g., [13, 14]), although they did not aimed at investigating the performance of PubMed in finding highly-related articles. The service can be a representative text-based inter-article similarity estimation technique in the biomedical domain, and it was shown to be one of the best systems in finding related biomedical articles [11]. It integrates several typical factors considered by many text-based similarity estimation techniques [6, 7]. The factors include (1) article length; (2) word recognition and stemming; (3) word positions (i.e., in certain key parts of the articles, such as the titles of articles); (4) key terms of articles in domain-specific thesauri (i.e., Medical Subject Headings, MeSH, available at http://www.ncbi.nlm.nih.gov/mesh); and (5) word weights (i.e. weighted by how frequently a word appears in the article and how rarely the word appears in the collection of articles). Therefore, with PubMed as a baseline, we can measure the practical contribution of PBC to the retrieval of related articles.

For the target article *r* of each gene-disease pair *z*, PubMed was asked (in November 2014) to recommend a sequence *C* of related articles for *r*. We removed from *C* all articles that are *not* candidate articles for *z*. As noted above, the candidate articles for *z* include highly related articles (judged by biomedical experts), as well as those near-miss articles that should not be highly related to *r*. Therefore, by focusing on the candidate articles in *C* we can compare PubMed and PBC more objectively. Moreover, those highly related articles that are not recommended by PubMed are appended to *C*, as these highly related articles are actually ranked lowest by PubMed.

### Evaluation criteria

We employ two criteria to evaluate the systems: *Mean average precision* (MAP) and average P@X. MAP is commonly employed to evaluate text rankers. It is defined in Eq 10, where |*Q*| is the number of gene-disease pairs (recall that each pair has an article designated as the target article), *k*_*i*_ is number of articles that are believed (by the experts) to be highly related to the target article for the *i* ^th^ gene-disease pair, and *Arc*_*i*_(*j*) is the number of articles whose ranks are higher than or equal to that of the *j* ^th^ highly related article for the *i* ^th^ gene-disease pair.

$$MAP=\frac{\sum_{i=1}^{\left|Q\right|}AP(i)}{\left|Q\right|},\;AP(i)=\frac{\sum_{j=1}^{k_{i}}\frac{j}{Arc_{i}(j)}}{k_{i}}$$ (10)

Therefore, *AP*(*i*) is actually the average precision (AP) for the *i* ^th^ gene-disease pair. Given a target article *r* for a gene-disease pair, if a system can rank higher those articles that are highly related to *r*, AP for the gene-disease pair will be higher. MAP is simply the average of the AP values for all gene-disease pairs.

On the other hand, average P@X measures how each system ranks highly related articles at the top-X positions. It is defined in Eq 11.

$$Average\;P@X=\frac{\sum_{i=1}^{\left|Q\right|}P@X(i)}{\left|Q\right|}$$ (11)

$$P@X(i)=\frac{For\;pair\;i,\;number\;of\;top-X\;articles\;that\;are\;highly\;related}{X}$$ (12)

In Eq 11, |*Q*| is the number of gene-disease pairs, and hence average P@X is the average of the P@X values for all gene-disease pairs. The P@X value for the *i* ^th^ gene-disease pair is defined in Eq 12, which measures the precision when top-X articles are seen by the reader. By setting X to a small value, P@X can indicate how a system ranks highly related articles very high. We thus report average P@X, with X set to 1, 3, and 5.

Moreover, to verify whether the performance differences between PBC and the baselines are *statistically significant*, we conduct two-sided and paired t-tests with 95% confidence level. The significance tests are conducted on the AP and P@X values of the systems on all the gene-disease pairs.

### Results in Experiment I

**Fig 3:**
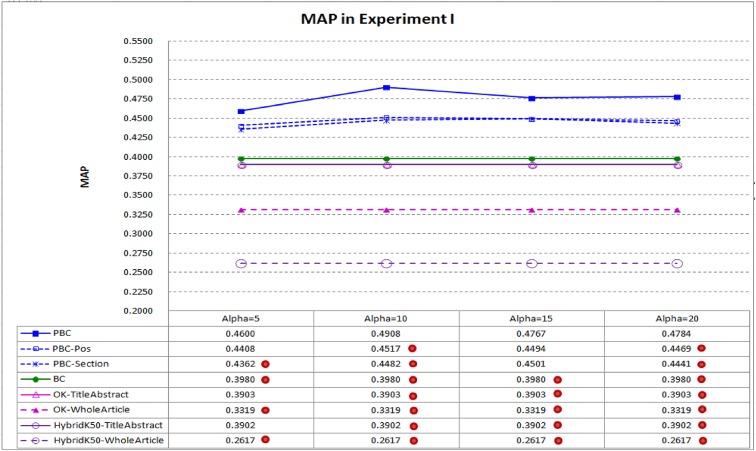
MAP in Experiment I. PBC performs better than BC and *HybridK50-TitleAbstract*, which are the best baselines. It achieves *significantly* better MAP under different settings of α (a dot on a system indicates that performance difference between the system and PBC is statistically significant).

Fig 3 compares MAP of PBC and seven baselines: the link-based baseline (BC), the two text-based baselines (*OK-TitleAbstract* and *OK-WholeArticle*), the two hybrid baselines (*HybridK50-TitleAbstract* and *HybridK50-WholeArticle*) and the two different settings of PBC (*PBC-Pos* and *PBC-Section*). PBC has better performance when the parameter α (alpha) is set to 10 to 20, which is close to the number of words in a sentence used to explain why a reference is cited, and hence properly setting α should not be a difficult task. PBC performs best, with its MAP *significantly* better than all baselines when α is set to 10 or 20 (recall that we conduct two-sided and paired t-tests with 95% confidence level to check whether performance differences are statistically significant). BC and *HybridK50-TitleAbstract* are the best baselines, however when α is set to 10, MAP of PBC is 23.32% better than that of BC (0.4908 vs. 0.3980) and 25.78% better than that of *HybridK50-TitleAbstract* (0.4908 vs. 0.3902). The results confirm the contribution of the context passages of important citations. Moreover, PBC performs better than both *PBC-Pos* and *PBC-Section* as well, indicating that the frequency-based importance (employed by PBC, ref. Eq 4) is more helpful than position-based importance and section-based importance (employed by *PBC-Pos* and *PBC-Section*, ref. Eq 7 and Eq 8) for the retrieval of the highly related articles.

It is interesting to note that both *OK* and *HybridK50* perform better when they work on titles and abstracts of the articles only (i.e., *OK-TitleAbstract* performs better than *OK-WholeArticle*, and *HybridK50-TitleAbstract* performs better than *HybridK50-WholeArticle*). The result confirms a common idea employed by many previous studies and search engines: certain key parts of the articles tend to be more informative. However, the key parts of an article *r* are often too short to express the core contents of *r*, which should include the research goals, research methods, and main findings of *r*.

**Fig 4:**
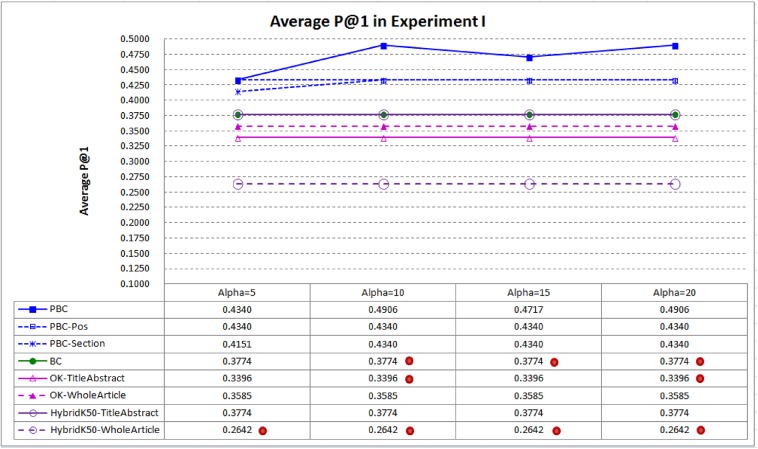
Average P@1 in Experiment I. PBC performs better than the best baselines BC and *HybridK50-TitleAbstract* in P@1, indicating that it is more capable in ranking the highly related articles at top–1 (a dot on a system indicates that performance difference between the system and PBC is statistically significant).

Fig 4 compares average P@1 of PBC and the baselines. PBC performs best, indicating that it is more capable in ranking the highly related articles at top–1. PBC performs *significantly* better than BC in average P@1 when α⊰{10, 15, 20}, however the performance differences (in average P@1) between PBC and *HybridK50-TitleAbstract* are *not* statistically significant. When α is set to 10, average P@1 of PBC is 29.99% better than that of BC (0.4906 vs. 0.3774), re-confirming the contribution of the context passages of important citations. As the average P@1 of PBC is 0.4906, PBC is able to rank the highly related articles at top–1 for about one half of the target articles. The performance result is thus encouraging in practice.

**Fig 5:**
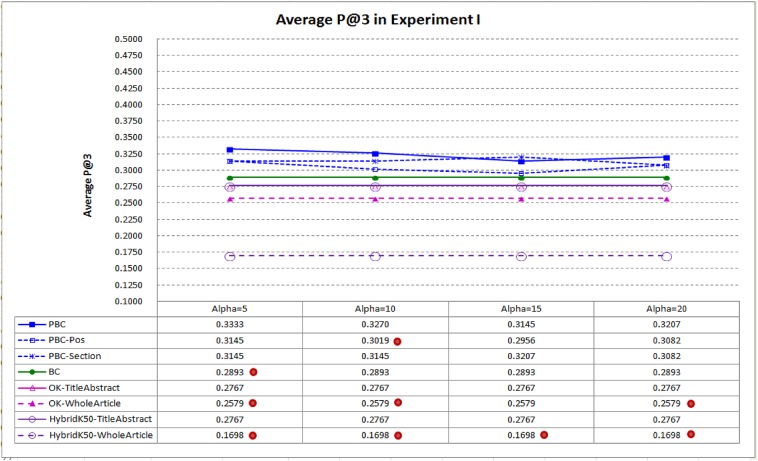
Average P@3 in Experiment I. PBC performs best, indicating that it is more capable in ranking the highly related articles at top–3 (a dot on a system indicates that performance difference between the system and PBC is statistically significant).

Fig 5 compares average P@3 of PBC and the baselines. Performance in average P@3 tends to be lower than performance in average P@1, as many target articles have less than three highly related articles. The result shows that PBC performs best again, indicating that it is more capable in ranking the highly related articles at top–3. When α is set to 10, average P@3 of PBC is 13.03% better than that of BC (0.3270 vs. 0.2893).

**Fig 6:**
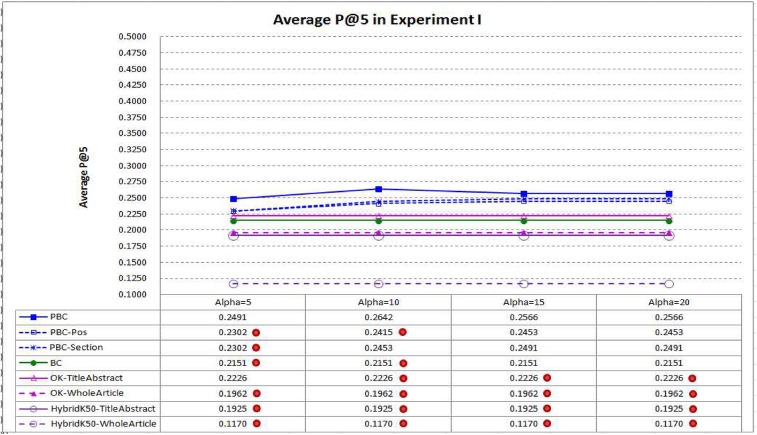
Average P@5 in Experiment I. PBC performs best, indicating that it is more capable in ranking the highly related articles at top–5 (a dot on a system indicates that performance difference between the system and PBC is statistically significant).

Fig 6 compares average P@5 of PBC and the baselines. PBC performs best again, indicating that it is more capable in ranking the highly related articles at top–5. It performs *significantly* better than BC in average P@5 when α⊰{5, 10}, as well as *HybridK50-TitleAbstract* under all settings of α. When α is set to 10, average P@5 of PBC is 22.83% better than that of BC (0.2642 vs. 0.2151).

By summarizing and analyzing the above results in Figs 3–6, we have the following findings:

1. PBC achieves the best performance by seamlessly integrating link-based component (i.e., bibliographic coupling similarity) with text-based component (i.e., important context passages of out-link citations). Performance differences between PBC and the baselines are *statistically significant* in many cases. Moreover, with the seamless integration, there is no need to tune the relative importance weights for the two components.
2. The parameter α (alpha) in PBC can be between 10 and 20. Although PBC performs significantly better than BC in MAP under all different settings for α⊰{5, 10, 15, 20} (recall Fig 3), it tends to perform better when α is between 10 and 20, which are close to the number of words that authors of biomedical articles employ to comment why a citation is discussed in the articles. Although the performance does not change dramatically when α⊰{10, 15, 20}, the experimental results show that α can be set to 10 for PBC and *PBC-Pos*; and 15 for *PBC-Section*. In the following experiments, we will report the performance of the systems with such individual better settings for them.
3. *PBC-Pos* cannot perform better than PBC. Although *PBC-Pos* performs better than BC, it cannot perform better than PBC. Position-based importance defined in Eq 7 cannot work well. A detailed analysis reveals the reason: less important citations may happen to be discussed at the places near to the end of an article (e.g., they are about the tools or methods employed by the article), while more important citations might be presented at the beginning of the article (e.g., key related work in literature review, which may be presented at the beginning of the article).
4. *PBC-Section* cannot perform better than PBC. Although *PBC-Section* performs better than BC, it cannot perform better than PBC. Section-based importance defined in Eq 8 cannot work well either. A detailed analysis identifies the reason: some articles do not have a specific section named ‘result’ and important citations may be discussed in sections other than the result section.
5. BC and the hybrid baseline *HybridK50-TitleAbstract* have quite similar performance. *HybridK50-TitleAbstract* performs somewhat better than BC in average P@1 and P@3, as it does not perform *significantly* worse than PBC in average P@1 and P@3 (ref. Figs 4 and 5). The result re-confirms the finding reported in [37]: *HybridK50* performed better than BC in certain cases.

As PBC integrates two components (link-based similarity and text-based similarity), we are interested in the relative contribution of each component. We implement two systems *PBC-link* and *PBC-text*, which respectively employ the link-based similarity and the text-based similarity but not both (i.e., *PBC-link* employs the upper part of Eq 3, while *PBC-text* employs the lower part of Eq 3). Table 2 summarizes the performance of the two components. PBC performs significantly better than both *PBC-link* and *PBC-text* in MAP and average P@5, indicating that integration of the two components is helpful. The result also shows that although *PBC-link* tends to perform better than *PBC-text*, their performance differences are *not* statistically significant in MAP and average P@X (X = 1, 3, and 5).

**Table 2:** Performance of the two components (*PBC-link* and *PBC-text*) of PBC.

	MAP	Average P@1	Average P@3	Average P@5
PBC	**0.4908**	**0.4906**	**0.3270**	**0.2642**
*PBC-link*	0.3966^[s]^	0.3962	0.2830	0.2226^[s]^
*PBC-text*	0.3763^[s]^	0.3396^[s]^	0.2516^[s]^	0.2075^[s]^

‘[*s*]’ on a system indicates that performance difference between the system and PBC is statistically significant.

**Fig 7:**
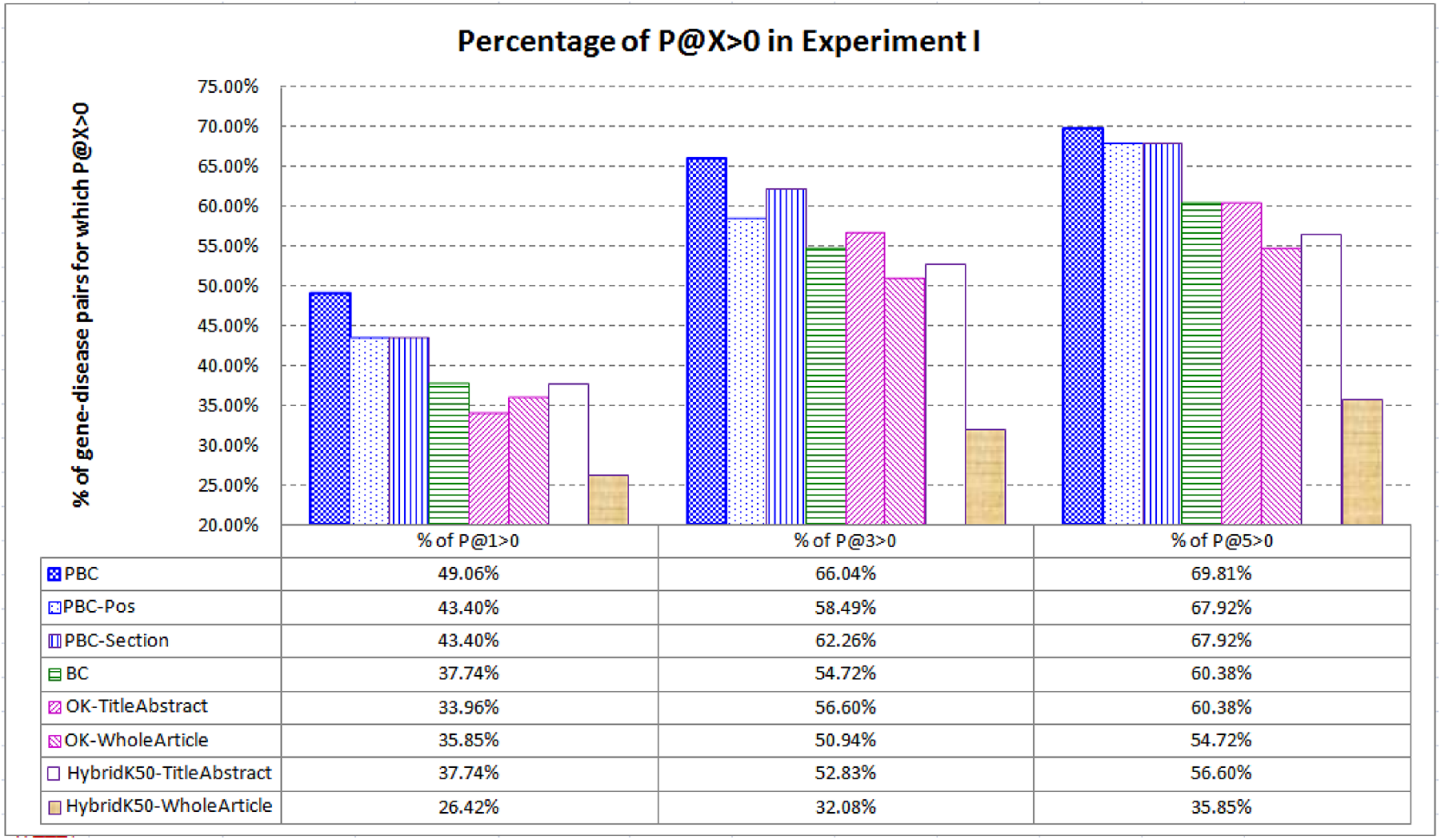
Percentage of the gene-disease pairs for which P@X > 0 in Experiment I. PBC ranks the highly related articles at top–1, top–3, and top–5 for a higher percentage of gene-disease pairs.

We are also interested in the *percentage* of the gene-disease pairs for which P@X > 0. Recall that each gene-disease pair has one article designated as the target article for which highly related articles are retrieved. Therefore, a higher percentage achieved by a system indicates that the system can help researchers to read a small number of recommended references for a higher percentage of the gene-disease pairs. Fig 7 shows the result (the systems employ their best settings for α: 10 for PBC and *PBC-Pos*; and 15 for *PBC-Section*). PBC ranks very high (top–3) the highly related articles for over 66% of the gene-disease pairs, while all the baselines cannot achieve the high percentage. The contribution is of practical significance to researchers, because there are a huge number of biomedical issues (e.g., associations among specific genes, chemicals, proteins, and diseases), and for each issue, there are often a large number of candidate articles to be checked by the researchers.

### Results in Experiment II

We further evaluate the practical contribution of PBC by comparing it with the “Related Citations” function provided by PubMed. Table 3 summarizes how PubMed retrieves related articles for the gene-disease pairs. Recall that among the articles recommended by PubMed, we focus on the candidate articles for each target article *r*, because the candidate articles have included those articles that are highly related to *r* (judged by biomedical experts), as well as those near-miss articles that should not be highly related to *r*. The result shows that PubMed does not recommend the highly related articles for over 56% (= 30/53) of the target articles, indicating that the service of retrieving related articles for a given biomedical article should be further improved.

**Table 3:** How does PubMed retrieve related articles for the gene-disease pairs. PubMed does not recommend the highly related articles for over 56% of the target articles, indicating that the service of retrieving related articles for a given biomedical article should be further improved.

	Are the highly related articles recommended by PubMed?	
	Yes	No
**Number of recommended candidate articles > 1**	14 (26.42%)	5 (9.43%)
**Number of recommended candidate articles ≤ 1**	9 (16.98%)	25 (47.17%)

**Fig 8:**
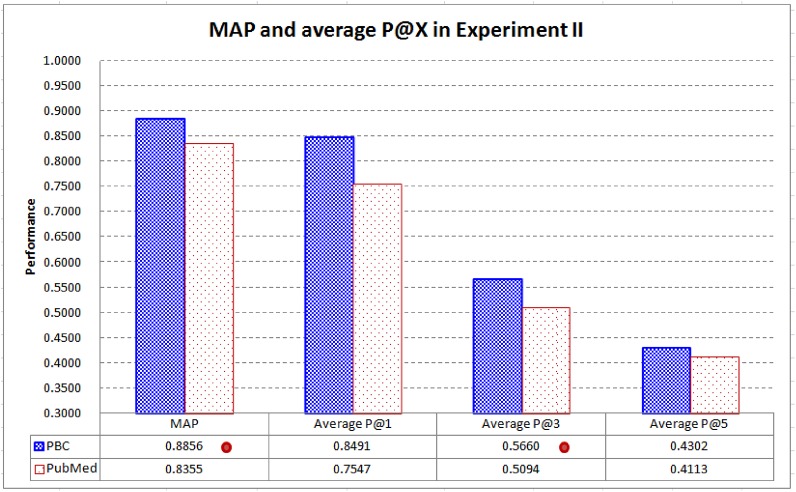
MAP and average P@X in Experiment II. PBC performs better than PubMed in MAP and average P@X (X = 1, 3, and 5), with *statistically significant* improvements in MAP and average P@3 (a dot on a system indicates that performance difference between PBC and PubMed is statistically significant).

Fig 8 compares the performance of PBC and PubMed in MAP and average P@X. PBC performs better than PubMed in MAP and average P@X (X = 1, 3, and 5), with *statistically significant* improvements in MAP and average P@3. MAP of PBC is 6% better than that of PubMed (0.8856 vs. 0.8355), while average P@3 of PBC is 11.11% better than that of PubMed (0.5660 vs. 0.5094). As PubMed is a representative and state-of-the-art text-based system to retrieve related biomedical articles [11], the results further confirm that text-based techniques are not necessarily a good choice for the retrieval of the highly related articles. PBC can be a significantly better technique to retrieve related articles without needing to employ complicated language processing techniques and thesauri.

**Fig 9:**
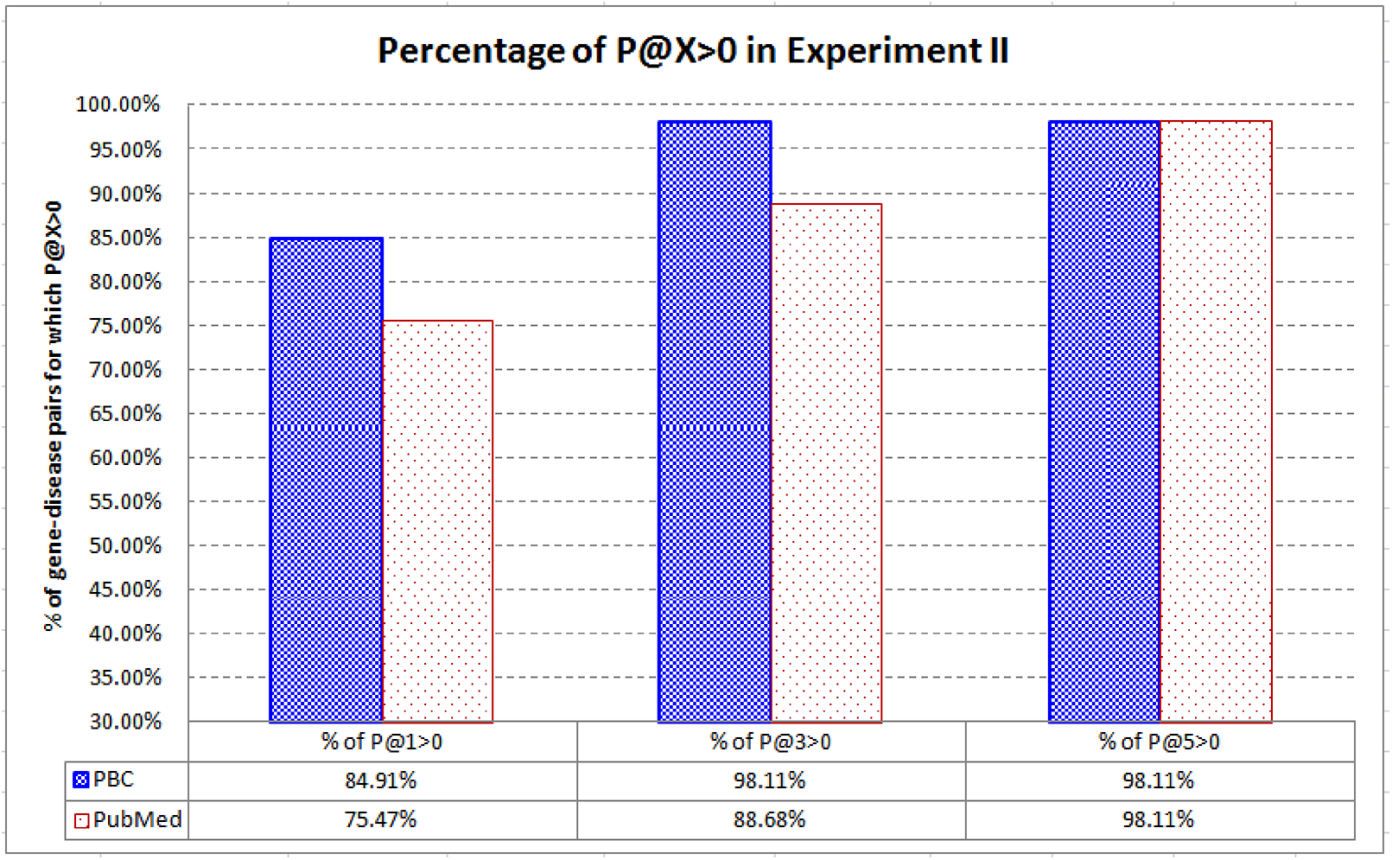
Percentage of the gene-disease pairs for which P@X > 0 in Experiment II. When compared with PubMed, PBC can rank the highly related articles at top–1 and top–3 for a higher percentage of the gene-disease pairs.

We are also interested in the *percentage* of the gene-disease pairs for which P@X > 0. Fig 9 shows the result. When compared with PubMed, PBC can rank the highly related articles very high (at top–1 and top–3) for a higher percentage of target articles of the gene-disease pairs. As a huge number of associations among biomedical entities are routinely analyzed by researchers and text mining systems, PBC is more capable in recommending the highly related articles for each association.

## Discussion

### Application and suggestion

The experimental results have shown that PBC performs *significantly* better than several linked-based, text-based, and hybrid techniques in recommending those articles that biomedical experts believed to be highly related to specific articles about gene-disease associations. Given an article, PBC can recommend the highly related articles to support more timely and complete cross validation of the evidence published in the articles. PBC can also serve as a front-end processor for text mining systems so that the systems can focus on the highly related articles, and hence efficiency and quality of the mining results may be improved.

PBC requires context passages as input, which are in the full texts of the articles. Many related studies have identified the contributions of the full texts (ref. *Related Work*), as the full texts can provide several kinds of helpful information, including proximity of citations in the articles [27–28], contents of the articles [24, 36–37], and citation passages in the articles [19, 29–35]. We re-confirm the contributions of the full texts. With the full-texts of the articles, PBC performs significantly better than those techniques that work on bibliography (i.e., bibliographic coupling) as well as titles and abstracts (i.e., *OK-TitleAbstract*, *HybridK50-TitleAbstract*, and PubMed) of the articles. However, in practice, ideas of PBC and these previous studies can be realized only for those articles whose full texts are publicly available (e.g., published in an open-access way). Therefore, traditional retrieval systems can be invoked to rank those articles whose full texts are not available, while PBC should be employed to rank full-text articles so that highly related articles can be recommended for cross-validation of highly related evidence published in literature.

We also suggest that PBC can be incorporated into biomedical search engines (e.g., PubMed), which have a database of full-text articles for which context passages of out-link citations can be pre-processed and cached for efficient estimation of inter-article similarity. For an article *d*, the preprocessing aims at identifying the citations in *d*, and for each citation *c*, identifying its context passage and degree of importance. The preprocessed and cached information makes the search engines able to efficiently identify related articles for a newly published article.

### Limitation and future research

The data collection strategy has a possible limitation. As noted in the section of *The data*, for each gene-disease pair <*g*, *d*> we collect two kinds of articles: (1) those articles that biomedical experts selected to annotate <*g*, *d*> (and hence they are highly related to each other), and (2) those “near-miss” articles that are *not* selected by the biomedical experts and only mention *g* or *d* but *not* both (and hence they should *not* be highly related to <*g*, *d*>). This data collection strategy is reasonable, and it provides convincing judgment of relatedness between articles so that we can conduct empirical evaluation objectively. However, we cannot perfectly exclude the possibility that some near-miss articles might still have a certain degree of relevance to the highly related articles. Moreover, the possible effect of those articles that are not included in the experimental data is not investigated.

PBC has technical limitations as well, which deserve tackling in future research. Out-link citations in an article are recognized by parsing the numbers and the hyperlinks of the citations in the article. This way of citation recognition can work well for those articles in which each citation is denoted by a reference number or a hyperlink. However, for those articles that are not in webpage form (e.g., in plain text or PDF form) and do not employ numbers to denote citations, more complicated ways to recognize the citations should be employed (e.g., designing templates, patterns, machine learning techniques to recognize the citations [44–45]). Moreover, the context passage of a citation in an article is recognized by extracting the words before the places where the citation is discussed in the article. This way of extracting context passages is efficient and shown to be helpful in retrieving highly related articles, indicating that the text used to comment a citation tends to appear before the citation. However, the context passage does not necessarily appear before the places, and a sentence might even contain several segments that are not related to the citation. More complicated linguistic patterns (e.g., [38]) may thus be employed. It is interesting to investigate whether the more complicated ways for citation recognition and passage extraction can further significantly improve the retrieval of the highly related articles.

Another potential extension for PBC is for the estimation of the degree of importance for each citation. We have tested three techniques to estimate the importance (i.e., the *frequency*-based, *position*-based, and *section*-based techniques) and find that the frequency-based technique performs best. However, we find that important citations in an article are not necessarily discussed more than two times in the article. It is thus interesting to investigate whether proper integration of the three techniques may significantly improve the retrieval of the highly related articles.

## Conclusion

Biomedical articles can be *highly related* to each other if they share similar core contents, including research goals, methods, and findings. In this paper, we present a technique PBC that given an article *r*, retrieves highly related articles for *r*, even when *r* is cited by very few (or even no) articles (e.g., *r* is recently published). Retrieval of such highly related articles is essential for researchers, who often need to carefully read multiple articles to exclude unproven or controversial evidence about specific issues (e.g., associations among biomedical entities, such as genes, chemicals, proteins, and diseases). Retrieval of the highly related articles is challenging as well, as it is quite difficult to recognize the core contents of the biomedical articles.

PBC considers the *out-link* citations (references) in articles to estimate inter-article similarity, and hence it can work well for the common case where articles are cited by very few (and even no) articles. Moreover, it seamlessly integrates bibliographic coupling with the information collected from *context passages* around *important* citations in the articles. Empirical evaluation on real-world data show that PBC performs significantly better than several representative techniques (including link-based, text-based, and hybrid techniques, as well as PubMed) in recommending highly related articles about gene-disease associations. PBC should thus be incorporated into biomedical search engines so that cross validation of biomedical evidence can be supported for authors, readers, reviewers, and text mining systems.

## Supporting Information

S1 File The PubMed Central (PMC) articles that are employed as the experimental data. There are 53 gene-disease association pairs. Each pair <*g*, *d*> has two kinds of articles: (1) those that biomedical experts selected to annotate the pair, and (2) those that mention *g* or *d* but *not* both. The former kind of articles are thus highly related to each other (and one of them is designated as the target article in the experiment), while the latter kind of articles should be not highly related to the target article from the perspective of <*g*, *d*>. For each candidate article *d* for a target article *r*, we also record eight similarity values, which are respectively produced by PBC and the seven baselines (*PBC-Pos*, *PBC-Section*, *BC*, *OK-TitleAbstract*, *OK-WholeArticle*, *HybridK50-TitleAbstract*, and *HybridK50- WholeArticle*).
(RAR)
