# Correction: Passage-Based Bibliographic Coupling: An Inter-Article Similarity Measure for Biomedical Articles

**DOI:** 10.1371/journal.pone.0142026

**Published:** 2015-10-29

**Authors:** The PLOS ONE Staff

## Notice of Republication

S1 File was published in error. The publisher apologizes for the error. The error was corrected in the HTML versions of this article on October 16, 2015, and the corrected file is available in the supporting information of this article.

## Supporting Information

S1 File The PubMed Central (PMC) articles that are employed as the experimental data. There are 53 gene-disease association pairs. Each pair <*g*, *d*> has two kinds of articles: (1) those that biomedical experts selected to annotate the pair, and (2) those that mention *g* or *d* but *not* both. The former kind of articles are thus highly related to each other (and one of them is designated as the target article in the experiment), while the latter kind of articles should be not highly related to the target article from the perspective of <*g*, *d*>. For each candidate article *d* for a target article *r*, we also record eight similarity values, which are respectively produced by PBC and the seven baselines (*PBC-Pos*, *PBC-Section*, *BC*, *OK-TitleAbstract*, *OK-WholeArticle*, *HybridK50-TitleAbstract*, and *HybridK50- WholeArticle*).
(RAR)
